# Great Things

**Published:** 1866-07

**Authors:** 


### GREAT THINGS.

Correspondence of Northern Ohio Dental Society.

Could we acquire greatness of conception and execution, at little cost in time and effort, there is not one among us who would not be great. And could we see that all greatness consists in goodness, fully one-half of the obstructions in the way of its attainment would disappear, as by the potent hand of magic.

It is a *great* thing to know that the greatest is made up of parts so small, as to escape the attention of him who is not willing to contemplate the least, and apparently most insignificant, infinitissimal portions; for upon the perfection of the structure and corelation of these, does the harmony of the whole depend.

These aphorisins are true throughout the broad domain of philosophy. And there is no department of the theoretical or practical work belonging to our profession, where this is not plainly apparent. Who are the best able to understand the working of the whole organism, if it be not those who have minutely studied all the varied little things that go to make it up?

He who is not fastidiously nice in his discriminations, can never be a complete and *safe* performer of the many varying operations required of him in the practice of dentistry. Hence, being a *slovenly* dentist or surgeon, is equivalent to being a *poor* dentist or surgeon.

If nature is so careful of the (naturally) invisible little cells out of which she builds the organs of the body, should we be ashamed to call optical instruments to our assistance, until these be revealed in clearest definition to our delighted gaze? Be assured, the time is not far distant, when he who is not conversant with minutest histology and microscopic anatomy, shall find it difficult to persuade even himself that he is entitled to be considered among the great things, without which the world could not continue!

Our profession, though but in the early bud of its usefulness, even now shadows forth the necessity of the broadest and deepest erudition, in all who aspire to be masters of its philosophy, and its endlessly varied detail of art. So, if you would be great, it will not do to tarry in outward, vague, and ambiguous diagnosis, and slovenly, superficial performance of the work undertaken; for these have been the *opprobrii*, that have hung like an incubus about the neck of the profession in the past, preventing its full developement, and the proper appreciation of its practitioners and patrons. Just so soon as those who enter the profession, shall determine to do so with a great mind, and high purpose, there will be no more half-way pupils or practitioners in our body! And just so soon as we properly appreciate ourselves, and our really high calling, the people will not be slow to seek only such as are capable of doing them, not temporary and doubtful good, but great benefit, lasting as life itself.

Many, now in the profession, of popular standing and ability of no mean measure, are keeping aloof from associations and colleges, for no better reason than that they are not great enough to ignore prejudice, and stem the tide of the bug-bear fears of "what will my patrons and the people say, if I thus confess my ignorance of things I should have known long ago?" The question is not one of popular reputution for ability, but of personal consciousness of real ability, which we can no longer ignore, in delaying to make ourselves equal to the increased demands upon us! He who is determined to be equal to the best, will not refuse the cost, in effort, time, and money—first to compare qualifications, impartially, at every opportunity, so that he may know what is and what is not sound and reliable, in doctrine and practice. And when the true standard is found, he will not rest till it is in process of active attainment, if not already in his possession.

Minds of this earnest stamp are never at a loss for occupation; for so soon as they have practicalized the well defined ideal conception, in their works, they have so sharpened their powers of perception, and, may be, invention also, as to take in a higher and more extended ideal upon which to busy themselves. And thus they go on in a blessed enthusiasm of attainment, from conquering their own darkness to conquer the difficulties that beset the path of beneficent work!

By a constant reputation of these processes, it is feasible to ultimately attain a height and power that would make us dizzy to attempt, at a single effort, even to contemplate. Compare dentistry of 25 years ago with that of to-day, and the wildest hopes for the future would seem to be justified!

If all great things are but aggregations of "little thlngs," how doth it become us to "forget not the assembling ourselves together, as the manner of some is," to give and to receive of the things attained and desirable to be attained, stimulating and being stimulated, into higher and higher ambition, for the great body of which each is an integral part, and without which it cannot be great or complete.

And now, permit me heartily to commend you to angel guidance, in the blessed work of head and heart, and of hand, immediately before you. As ever,

ATKINSON.

